# Mycovirus Vector‐Mediated RNAi for Effective Gene Knockdown in Pine Wood Nematodes

**DOI:** 10.1111/pbi.70567

**Published:** 2026-02-09

**Authors:** Ruiling Bian, Yifan Zhang, Zhihao Zhang, Yuchi Bai, Peiqin Li, Guanghui Tang, Lihua Guo, Huan Liu, Ida Bagus Andika, Qiaoxia Shang, Liying Sun

**Affiliations:** ^1^ State Key Laboratory of Crop Stress Biology for Arid Areas and College of Plant Protection Northwest A&F University Yangling China; ^2^ Institute of Future Agriculture Northwest A&F University Yangling Shaanxi China; ^3^ College of Forestry Sciences Northwest A&F University Yangling Shaanxi China; ^4^ State Key Laboratory for Biology of Plant Diseases and Insect Pests, Institute of Plant Protection Chinese Academy of Agricultural Sciences Beijing China; ^5^ School of Modern Agriculture and Biotechnology Ankang University Ankang Shaanxi China; ^6^ College of Bioscience and Resource Environment, Key Laboratory for Northern Urban Agriculture of Ministry of Agriculture and Rural Affairs Beijing University of Agriculture Beijing China

**Keywords:** mycovirus, nematodes, RNA interference, virus‐induced gene silencing

Pine wilt disease is caused by the nematode *Bursaphelenchus xylophilus*. *B. xylophilus* spreads via the beetle *Monochamus* alternatus, which creates entry points for *B. xylophilus* to feed on pine resin and endophytic fungi. The nematode has reproductive and dispersal forms. Juvenile nematodes reach the beetle's pupal chambers and spread when the beetle feeds on trees (Futai 2013).

RNA interference (RNAi), a gene knockdown technique, has been adapted to plant‐parasitic nematodes via soaking in dsRNA solution, but RNAi effects are limited and transient (Park et al. 2008). In contrast, feeding methods using fungi or plants expressing dsRNA offer more durable gene silencing. For *B. xylophilus*, which feeds on fungi, engineered fungi expressing dsRNA provide an effective gene‐silencing tool (Zhang et al. 2025). Virus‐induced gene silencing (VIGS) is a technique widely used to produce siRNAs. Mycoviruses like Fusarium graminearum gemytripvirus 1 (FgGMTV1) can induce VIGS in fungi through the accumulation of siRNAs (Zhang et al. 2023; Wang et al. 2022). Herein, we engineered *Fusarium graminearum* strains by transfecting them with FgGMTV1‐based vectors carrying essential nematode genes and used them to feed *B. xylophilus*.

To evaluate the efficacy of this system, we selected three target genes (*BxISP‐1*, *BxNDUF‐7*, and *BxNUO‐6*), based on their homology to *Caenorhabditis elegans CeISP‐1*, *CeNDUF‐7*, and *CeNUO‐6* encoding conserved components of mitochondrial respiratory chain complexes, for which RNAi knockdown causes lethality (Rauthan et al. 2015; Yang and Hekimi 2010). Although conserved across nematode species, they share low sequence similarity with their homologues in *F. graminearum* (Figures S1 and S2). Fragments of the target genes (GFP as a control) were cloned into FgGMTV1 vector (Figure 1a; methods in Appendix S1). These constructs were introduced into the *F. graminearum* with deletion in a gene encoding ALG‐2‐interacting protein X (ALIX, endosomal sorting protein). This fungal mutant exhibits a whitish phenotype, which enables better observation of the feeding nematodes. Juveniles of *B. xylophilus* were fed fungal strains harbouring FgGMTV1 vector. After two or more days of feeding, nematodes were collected for analysis (Figure 1b). PCR analysis confirmed infection of FgGMTV1 carrying each target gene fragment, and no colony growth difference on PDA was observed (Figure 1c,d; Figure S3), consistent with latent infection of FgGMTV1 (Li et al. 2020). For comparison, a dsRNA soaking assay was conducted using dsRNAs expressed in bacteria (Figure 1e). Nematodes were soaked in the dsRNA solutions for 24 h, then transferred onto virus‐free fungal plates for an additional 2 or more days of incubation. The collected nematodes were subsequently examined under identical conditions to assess morphology changes and the target genes. Similar phenotypic changes were observed in nematodes following FgGMTV1‐ and dsRNA soaking‐induced RNAi treatments. Compared to the control, nematodes subjected to BxISP‐1 knockdown were significantly larger, while those with BxNDUF‐7 knockdown showed developmental arrest (Figure 1f). BxNUO‐6 silencing group showed no morphological abnormalities (Figure 1f). The morphological changes observed were quite homogenous in both treatments with dsRNA and FgGMTV1‐carrying fungi, suggesting the similarly high transformation efficiency for both methods. RT‐qPCR analysis confirmed that both gene knockdown methods effectively suppressed the accumulation of target gene transcripts at 2 days after treatment, suggesting that FgGMTV1 can induce RNAi in a manner comparable to the dsRNA soaking method during the early stages (Figure 1g). However, by 7 days, only the FgGMTV1‐mediated method remained effective, with target gene transcripts still suppressed (Figure 1g). Both gene knockdown approaches led to a significant reduction in nematode numbers compared to the control at 2 and 7 days post‐treatment, except for BxISP‐1 knockdown mediated by FgGMTV1 at 2 days (Figure 1h), indicating that knockdown of the target genes was deleterious for nematode viability. Thus, for BxNUO‐6 silencing, the induced physiological changes likely did not manifest as clear phenotypic differences. Notably, by day 10, low numbers of nematodes remained in the dsRNA‐treated group, whereas in the FgGMTV1‐treated group, nematodes have almost entirely died (Figure 1h). The presence of dsRNA was confirmed in *B. xylophilus* after soaking with dsRNA fragments, but not after feeding on the fungus (Figure S4). FgGMTV1 is a circular single‐stranded DNA virus which, unlike ssRNA viruses, lacks a dsRNA intermediate stage during its replication. Thus, in the case of the FgGMTV1‐fungus system, the RNAi effect on the nematode is most likely mediated by the production of siRNAs, which are then transferred to *B. xylophilus* via feeding. Overall, these findings demonstrate that fungal virus‐mediated RNA silencing provides a more potent and persistent gene knockdown than the conventional soaking methods.

**FIGURE 1:**
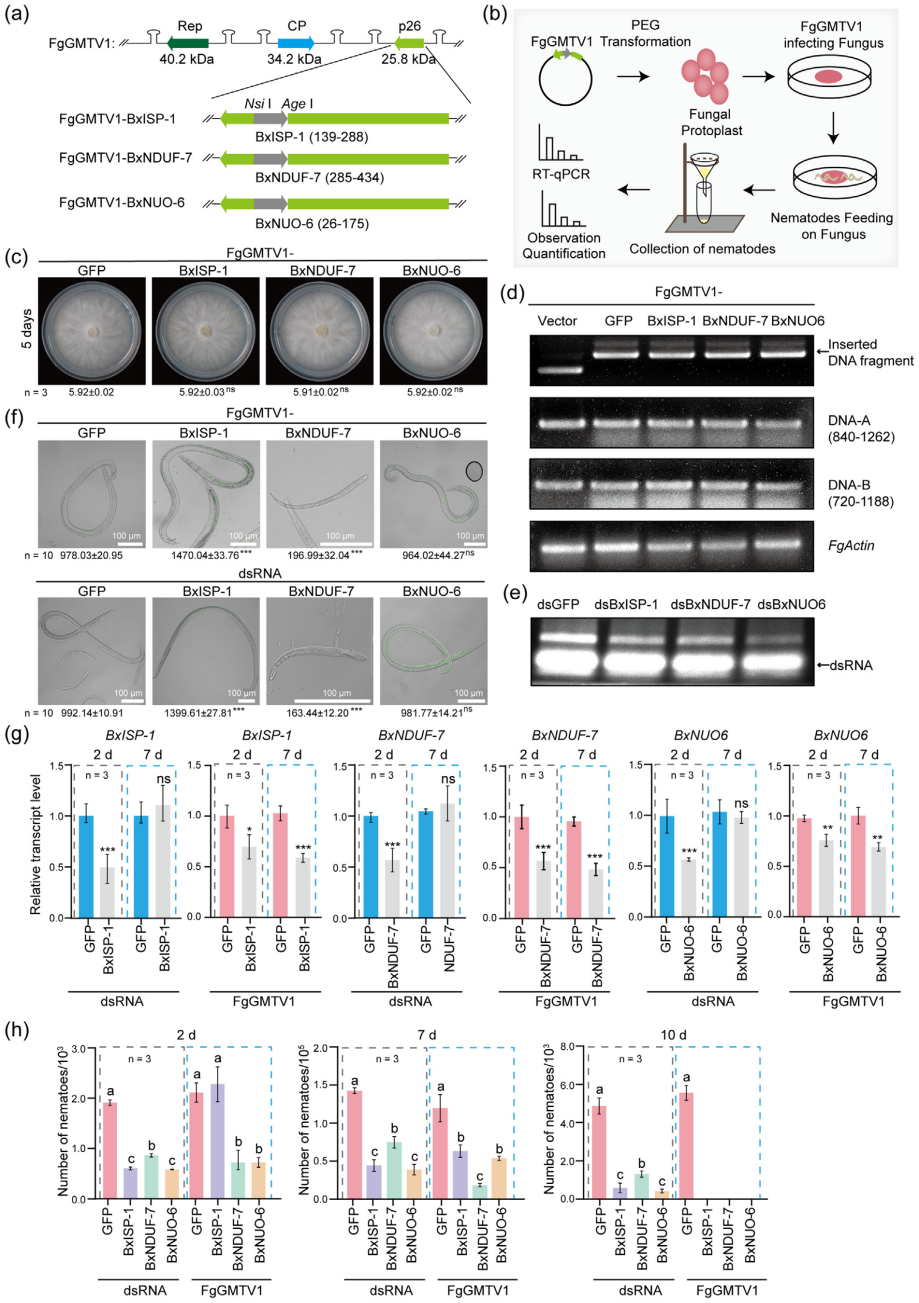
FgGMTV1‐fungus system mediates RNAi in *B. xylophilus*. (a) Diagram of the FgGMTV1 vector with fragments of the target genes. (b) RNAi experimental workflow. (c) Fungal colony morphology on PDA, with colony sizes (cm). Asterisks indicate significance (*p < 0.05, **p < 0.01, ***p < 0.001; Student's *t*‐test). (d) PCR detection of *B. xylophilus* target gene fragments and FgGMTV1 in infected fungi. (e) Gel electrophoresis of dsRNA of target genes. (f) Phenotypic observations of *B. xylophilus* after feeding on FgGMTV1‐infected fungi or dsRNA treatment, with measured nematode body length (μm). (g) Relative transcript expression of target genes. (h) Quantification of *B. xylophilus* individuals after silencing the target genes. Different letters indicate significant differences (*p* < 0.05, one‐way ANOVA, Tukey's HSD test).

The dsRNA soaking method delivers high concentrations of dsRNA directly to the nematodes, resulting in a stronger initial gene silencing effect. In contrast, the FgGMTV1‐fungus system can continuously supply siRNAs to the nematodes after feeding. Thus, unlike the dsRNA soaking method, whose effects are transient, this mycovirus‐mediated system provides a more robust and persistent gene knockdown.

Fungal‐mediated RNAi approaches relied on integrating transgenes encoding hairpin RNA into the fungal genomes for production of siRNAs, which are ingested by feeding nematodes to silence target genes. FgGMTV1‐fungus system operates on a similar principle, but it is easier to construct, making it suitable for high‐throughput gene screening. Moreover, it has potential as an alternative control measure for crop‐parasitic nematodes. Further investigation into suitable mycovirus‐fungus combinations for this system, along with a comprehensive environmental risk assessment, will pave the way for its application in the field.

## Author Contributions

R.B., Y.Z., and Z.Z. authors contributed equally to this work. L.S., R.B. and Q.S. designed research; R.B., Y.Z., Z.Z., Y.B., P.L., H.L., G.T. and L.G. performed research; R.B., Y.Z. analysed data and R.B., Y.Z., I.B.A. and L.S. wrote the manuscript.

## Funding

This research was funded by the Interdisciplinary Frontier Innovation Team Program of Northwest A&F University (A1080524002) to LS, and Open Fund of State Key Laboratory of Agricultural and Forestry Biosecurity (SKL2025006) to YZ.

## Conflicts of Interest

The authors declare no conflicts of interest.

## Supporting information


**Appendix S1:** Materials and Methods, Supplementary Figures S1–S4 and Table S1.

## Data Availability

The data supporting the findings of this study are available in the S1.
