# Supplementary material for: Mycovirus Vector‐Mediated RNAi for Effective Gene Knockdown in Pine Wood Nematodes
Source: Plant Biotechnol J. 2026 Feb 9;24(6):3531–3. doi: 10.1111/pbi.70567 (PMC13205871; doi:10.1111/pbi.70567)
Supplement: Supplementary file 1 — Appendix S1: Materials and Methods, Supplementary Figures S1–S4 and Table S1. [file PBI-24-3531-s001.pdf]

Supplemental Information Appendix S1. Materials and Methods, Supplementary figures (Figure 1–4) and table (Table 1).

Mycovirus vector-mediated RNAi for effective gene knockdown in Pine Wood Nematodes

Ruiling Bian[#a,b], Yifan Zhang[# a,b], Zhihao Zhang[#a], Yuchi Bai[a], Peiqin Li[c], Guanghui Tang[c], Lihua Guo[d], Huan Liu[e], Ida Bagus Andika[a], Qiaoxia Shang*[f], Liying Sun*[a, b]

[a] State Key Laboratory of Crop Stress Biology for Arid Areas and College of Plant Protection, Northwest A&F University, 712100 Yangling, China

[b] Institute of Future Agriculture, Northwest A&F University, Yangling 712100, Shaanxi, China

[c] College of Forestry Sciences, Northwest A&F University, Yangling 712100, Shaanxi, China

[d] State Key Laboratory for Biology of Plant Diseases and Insect Pests, Institute of Plant Protection, Chinese Academy of Agricultural Sciences, Beijing 100081, China

[e] School of Modern Agriculture and Biotechnology, Ankang University, Ankang, Shaanxi, China

[f] College of Bioscience and Resource Environment, Key Laboratory for Northern Urban Agriculture of Ministry of Agriculture and Rural Affairs, Beijing University of Agriculture, Beijing 102206, China

Materials and Methods

Fungal strains and culture conditions

F. graminearum PH-1 mutant containing deletion in *FgALIX* gene (FGSG_05329) was generated through homologous recombination using DNA constructs containing a neomycin resistance cassette and sequences flanking the *FgALIX* locus, assembled via double-joint PCR (Yu et al. 2004). The recombinant DNA fragments were introduced into *F. graminearum* protoplasts by polyethylene glycol (PEG)-mediated transformation, following previously described protocols (Hou et al. 2002). The fungus and virus infected strains generated in this study were routinely cultured on potato dextrose agar (PDA) plates at 25 °C for 3-7 days for DNA preparation and morphological observation.

Cultivation of *B. xylophilus*

The *B. xylophilus* nematode isolate used in this study was collected from the Qinling Mountains Shaanxi, China (Sun et al. 2024). The *B. xylophilus* were feed on fungal culture plates and incubated in the dark at 25 °C.

Construction of recombinant FgGMTV1 vectors

To generate the VIGS plasmid constructs, gene fragments *BxISP-1* (nucleotide positions 139-288; Gene ID: BXYJ5.040170500), *BxNDUF-7* (285-434; Gene ID: BXYJ5.010150100), *BxNUO-6* (26-175; Gene ID: BXYJ5.010116800), were amplified from the total RNA of *B. xylophilus* using RT-PCR, based on sequences obtained from WormBase (https://parasite.wormbase.org/index.html). These amplified DNA fragments were inserted into an infectious clone plasmid derived from FgGMTV1, which had been digested with *Nsi* I and *Age* I, resulting in the recombinant constructs of FgGMTV1 carrying approximately 150 bp gene fragments of *BxISP-1*, *BxNDUF-7*, *BxNUO-6*, respectively (Zhang et al. 2023). The GFP gene fragment served as a control. All primers used in this study are listed in Supplementary Table S1.

Transfection of FgGMTV1 constructs

The recombinant FgGMTV1 constructs were then transfected into *F. graminearum* protoplast following the protocol described previously (Bian et al. 2020). Fungal genomic DNA was extracted using the CTAB method and subjected to PCR confirmation using FgGMTV1 specific primers (FgGMTV1-212-F/R) listed in Table S1, as previously described by (Jiang et al. 2020). The results indicated a positive transformation rate of approximately 30%~50%. The confirmed transformants

were subsequently transferred to fresh PDA plates for subculturing and propagation. DNA extracted from the subcultured isolates also yielded positive amplification signals when tested with the same virus-specific primers, confirming the stable maintenance of the viral construct.

Feeding *B. xylophilus* on FgGMTV1-infected fungi

Fungal strains infected with FgGMTV1 are cultured on PDA plates. Once the mycelium reaches approximately 1 cm from the edge, approximately 2000 second-stage *B. xylophilus* juveniles are placed on the central region of fungal mat. At 2, 7, and 10 days post-inoculation, nematodes are collected using the Baermann funnel method, as described previously (Tintori et al. 2022).

Generation of dsRNA

The GFP, BxISP-1, BxNDUF-7, and BxNUO6 gene fragments were cloned into the dsRNA expression plasmid L4440 (Belongbio, China), which was digested with *Sma* I and *Nco* I. The resulting plasmids were transformed into HT115 (DE3) competent cells（Biomed, China）using standard heat shock transformation protocols (Tenllado et al. 2003). A single colony was inoculated into 5 mL of LB with ampicillin (50 μg/mL), cultured at 37 ℃ with shaking at 200 rpm until the OD_{600} reached 0.4–0.8. The culture was scaled up transferring 4 mL into 400 mL of LB, and IPTG was added to 1 mM to produce dsRNA at 37 ℃ for 5 h. dsRNA was extracted using the TransZol Up Plus RNA Kit (TransGen, China) following the manufacturer's instructions and verified by 1% agarose gel electrophoresis.

Fluorescence signal imaging and body length measurement of *B. xylophilus*

Phenotypic observations of *B. xylophilus* were carried out after feeding on FgGMTV1-infected fungi or after soaking in dsRNA solution for 48 hours, followed by feeding on wild-type fungi for 2 days. For anesthetizing nematodes, a 0.25 M levamisole hydrochloride solution was prepared with distilled water. Nematodes were washed 2–3 times with sterile water to remove impurities (Chen et al. 2022). Approximately 50–100 nematodes were placed on a slide with 10 μL of levamisole, then covered gently to avoid compression. After 10–20 minutes at room temperature, phenotypes were observed under a confocal microscope. Autofluorescence was excited at 488 nm and captured at 510–550 nm. Nematode body length was measured using Image J software (version 1.54f, National Institutes of Health, USA). Ten nematodes were measured for each treatment, and standard deviations were calculated using the STDEV.S function in Excel.

Quantification of *B. xylophilus* and data analysis

The collected *B. xylophilus* samples were thoroughly mixed by pipetting and incubated at 4 °C for 5 minutes to reduce nematode movement. A 10 μL aliquot was evenly spread on an agar plate and examined under a stereomicroscope (~4.5×magnification). Nematodes were manually counted with a hand-held counter. Each treatment was performed in triplicate. For statistic analysis, one-way ANOVA followed by followed by Tukey's Honest Significant Difference (HSD) post hoc test was conducted using SPSS version 26.0 (IBM Corp., Armonk, NY, USA), with significance set at $P<0.05$, following the methodology described by Vandepitte et al. (Vandepitte et al. 2014).

Total RNA extraction from *B. xylophilus* and RT-qPCR analysis

B. *xylophilus* were collected on days 2, 7, and 10 and thoroughly ground using a glass pestle directly within the tube to ensure complete homogenization. Total RNA was extracted using the Direct-zol™ RNA Miniprep Kit (ZYMO RESEARCH, USA). First-strand cDNAs were synthesized with EasyScript Reverse Transcriptase (TransGen, China). RT-qPCR was performed with Green qPCR SuperMix (TransGen, China) on a Gentier 96E system (TIANLONG, China). Gene expression was calculated using the $2^{\wedge-\Delta\Delta Ct}$ method, with three biological replicates. *BxActin* served as the internal reference gene (Qiu et al. 2013).

Detection of dsRNA in *B. xylophilus*

Total RNA extracted from *B. xylophilus* was divided into two aliquots, one treated with RNase I_f (specifically degrades ssRNA) and the other left untreated. Total RNA was incubated with RNase I_f (New England Biolabs, Ipswich, MA, USA) according to the supplier's recommendations at 37 °C for 10 min to remove single-stranded RNA. The reaction was subsequently terminated by heating at 70 °C for 20 min. The resulting RNase I_f-treated RNA sample was used for subsequent cDNA synthesis. Both RNA samples were used as templates for first-strand cDNA synthesis. For each reaction, RNA was pre-incubated at 95 °C or 68 °C for 5 min for denaturation, and immediately cooled on ice. Gene-specific primers were used for reverse transcription with EasyScript Reverse Transcriptase (TransGen, China), following the manufacturer's instructions. The resulting cDNA was then used as a template for PCR amplification using mixed DNA polymerase (CWBIO, China) as described above to detect dsRNA-derived transcripts (Wang et al. 2018).

Phylogenetic analysis

Amino acid sequences of *B. xylophilus* ISP-1, NDUF-7, and NUO-6 homologs (Clade IV) and homologs from other representative nematode species, including *Meloidogyne incognita* (Clade IV),

Brugia malayi (Clade III), *Trichinella spiralis* (Clade I), *Trissonchulus latispiculum* (Clade II), as well as *Arabidopsis thaliana* were retrieved from the WormBase database (https://parasite.wormbase.org/index.html) and Arabidopsis database (https://www.arabidopsis.org/). Phylogenetic trees were constructed separately for each protein. The trees were built using the Neighbor-Joining method in MEGA-X software, and branch support was assessed with 1,000 bootstrap replicates.

Reference

Bian, R., Andika, I., Pang, T., Lian, Z., Wei, S., Niu, E., Wu, Y., et al. (2020). "Facilitative and synergistic interactions between fungal and plant viruses". Proc Natl Acad Sci U S A, 117:3779-3788.

Chen, J., Hao, X., Wang, B., Ma, L.(2022). "Transcriptomics and coexpression network profiling of the effects of levamisole hydrochloride on *Bursaphelenchus xylophilus*". Pestic Biochem Physiol, 181:105019.

Hou, Z., Xue, C., Peng, Y., Katan, T., Kistler, H. C., Xu, J. R. (2002). "A mitogen-activated protein kinase gene (MGV1) in *Fusarium graminearum* is required for female fertility, heterokaryon formation, and plant infection". Mol Plant-Microbe Interact, 15(11), 1119–1127.

Jiang, C., Hei, R., Yang, Y., Zhang, S., Wang, Q., Wang, W., Zhang, Q., et al. (2020). "An orphan protein of Fusarium graminearum modulates host immunity by mediating proteasomal degradation of TaSnRK1α". Nat Commun, 11:4382.

Qiu, X., Wu, X., Huang, L., Tian, M., Ye, J. (2013). "Specifically expressed genes of the nematode *Bursaphelenchus xylophilus* involved with early interactions with pine trees". PLoS One, 8:e78063.

Sun, M., Liang, C., Fu, X., Liu, G., Zhong, Y., Wang, T., Tang, G., et al. (2024). "Nematocidal activity and biocontrol efficacy of endophytic Bacillus velezensis Pt-RP9 from Pinus tabuliformis against pine wilt disease caused by *Bursaphelenchus xylophilus*". Biol Control, 196:105579.

Tenllado, F., Martínez-García, B., Vargas, M., Díaz-Ruíz, J. (2003). "Crude extracts of bacterially expressed dsRNA can be used to protect plants against virus infections". BMC Biotech, 3:3.

Tintori, S., Sloat, S., Rockman, M. (2022). "Rapid Isolation of Wild Nematodes by Baermann Funnel". JoVE -J Vis Exp,

Vandepitte, K., De Meyer, T., Helsen, K., Van Acker, K., Roldán-Ruiz, I., Mergeay, J., Honnay, O. (2014). "Rapid genetic adaptation precedes the spread of an exotic plant species". Mol Ecol, 23:2157-2164.

Wang, P., Schulenberg, G., Whitlock, S., Worden, A., Zhou, N., Novak, S., Chen, W. (2018). "RNase If -treated quantitative PCR for dsRNA quantitation of RNAi trait in genetically modified crops". BMC Biotechnol, 18(1), 3.

Yu, J., Hamari, Z., Han, H., Seo, A., Reyes-Domínguez, Y., Scazzocchio, C. (2004). "Double-joint PCR: a PCR-based molecular tool for gene manipulations in filamentous fungi". FUNGAL GENET BIOL, 41(11), 973–981.

Zhang, L., Wang, S., Ruan, S., Nzabanita, C., Wang, Y., Guo, L. (2023). "A Mycovirus VIGS Vector

Confers Hypovirulence to a Plant Pathogenic Fungus to Control Wheat FHB". Adv Sci, 10:e2302606.

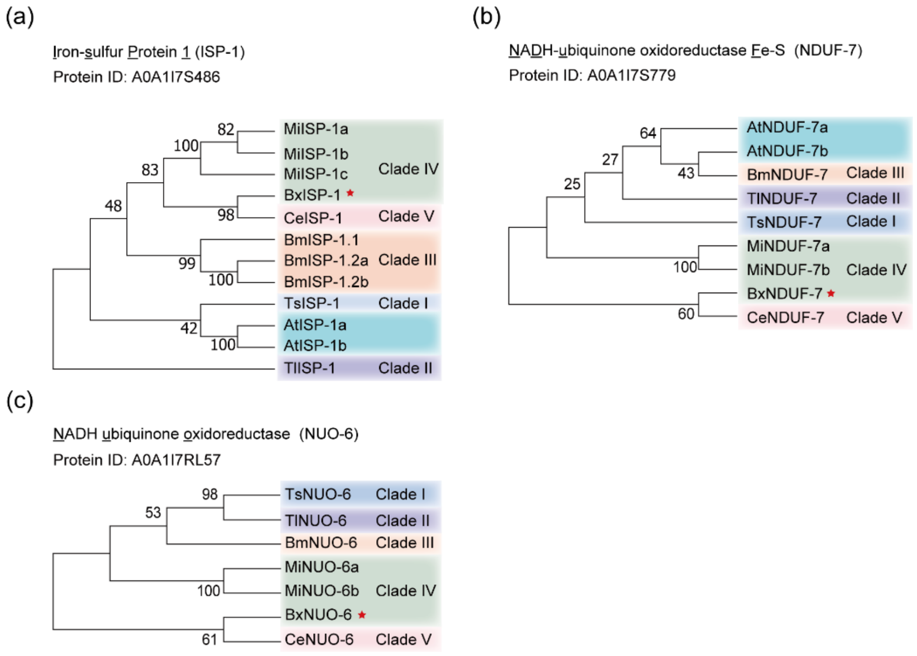

Figure S1. Phylogenetic analysis of the BxISP-1, BxNDUF-7, and BxNUO-6

(a-c) Phylogenetic analysis of the ISP-1, NDUF-7, and NUO-6 genes from *B. xylophilus* (Clade IV), *Meloidogyne incognita* (Clade IV), *C. elegans* (Clade V), *Brugia malayi* (Clade III), *Trichinella spiralis* (Clade I), *Trissonchulus latispiculum* (Clade II), and *Arabidopsis thaliana* based on amino acid sequences (MiISP-1a: A0A914LVP4, MiISP-1b: A0A914M4W4, MiISP-1c: A0A914N0J2, BxISP-1: A0A1I7S486, CeISP-1: O44512, BmISP-1.1: A0A0H5SC15, BmISP-1.2a: A0A0J9Y0K8, BmISP-1.2b: A0A0K0JCW6, TsISP-1: A0A0V1C321, AtISP-1a: AT5G13430.1, AtISP-1b: AT5G13440.1, TlISP-1: QYR20_01875000), (AtNUDF-7a: AT5G11770.1, AtNUDF-7b: ATCG00430.1, BmNUDF-7: A0A0K0JMG4, TlNDUF-7: QYR20_00855500, TsNUDF-7: EFV53373, MiNUDF-7a: A0A914LKQ5, MiNUDF-7b: A0A914KVX8, BxNDUF-7: A0A1I7S779, CeNDUF-7: Q94360), (TsNUO-6:E5RZ82, TlNUO-6: QYR20_02817600, BmNUO-6:A0A0J9Y791, MiNUO-6a:A0A914M3D9, MiNUO-6b:A0A914KJ95, BxNUO-6:A0A1I7RL57, CeNUO-6:Q23098). The phylogenetic trees were constructed using using the Neighbor-Joining method. Branch support was assessed with 1,000 bootstrap replicates.

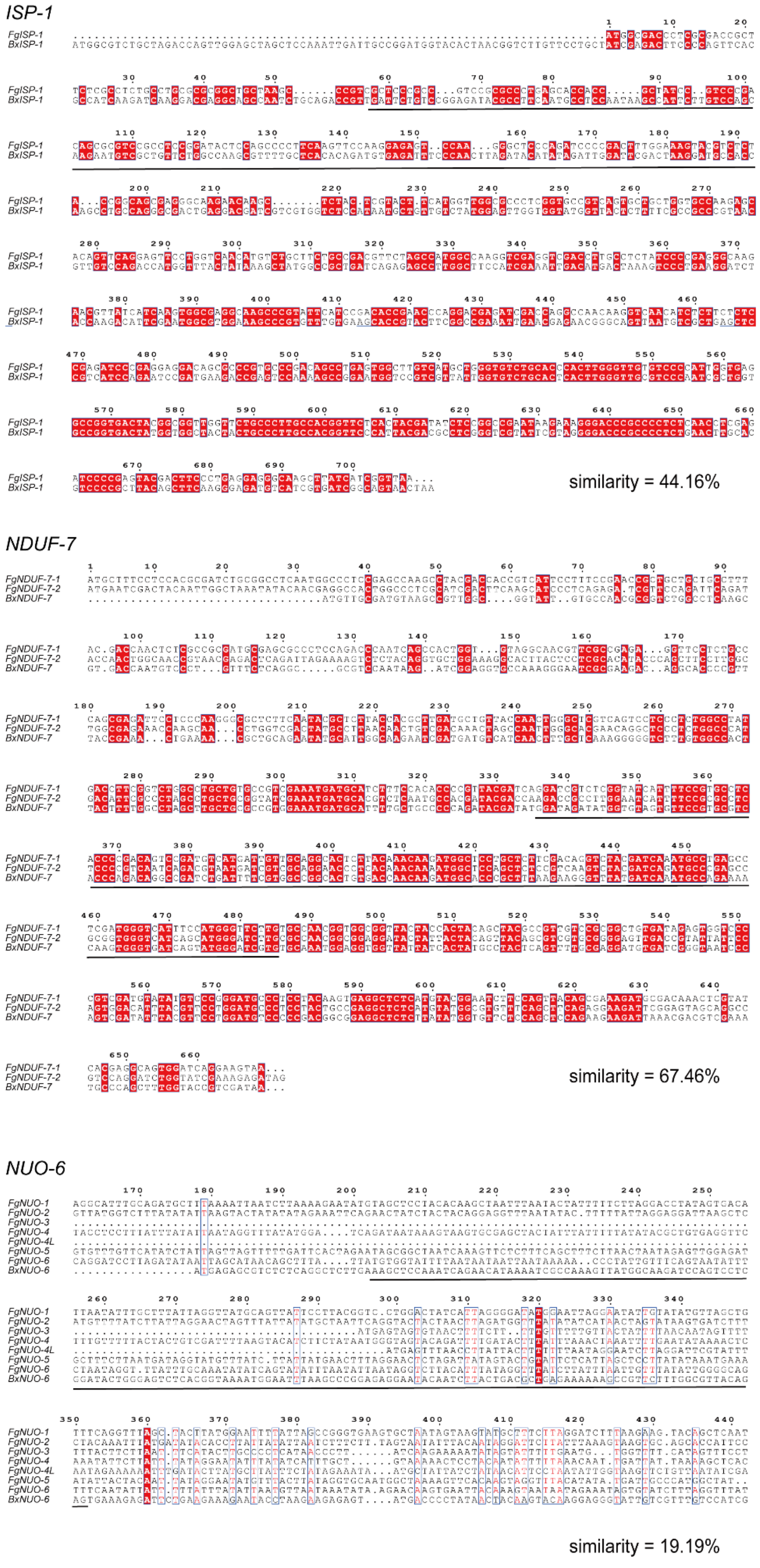

Figure S2: Nucleotide sequence alignment of *ISP-1*, *NDUF-7*, *and NUO-6* homologous genes

between *B. xylophilus* *and* *F. graminearum.* *(BxISP-1: BXYJ5.040170500, FgISP-1: XM_011325460) (BxNDUF-7: BXYJ5.010150100, FgNDUF-7-1: XM_011320929, FgNDUF-7-2: XM_011330120) (BxNUO-6: BXYJ5.010116800, FgNUO-1: YP_001249337, FgNUO-2: YP_001249299, FgNUO-3: YP_001249304, FgNUO-4: YP_001249339, FgNUO-4L: YP_001249312, FgNUO-5: YP_001249314, FgNUO-6: YP_001249346).* The black lines indicate sequences of gene fragments utilized in the construction of the VIGS vectors.

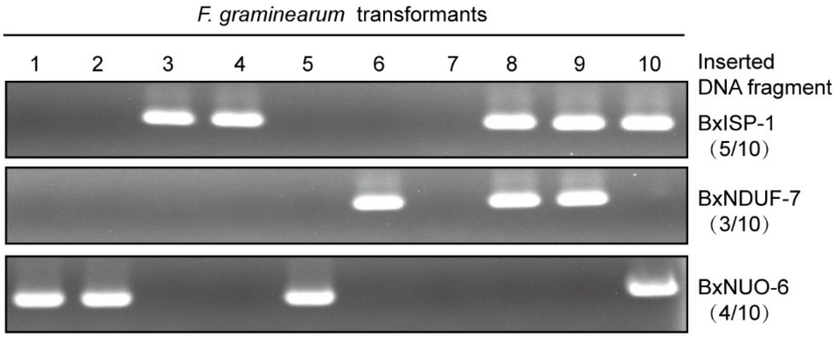

Figure S3. PCR detection of FgGMTV1 in regenerated *F. graminearum* strains after protoplast transfection.

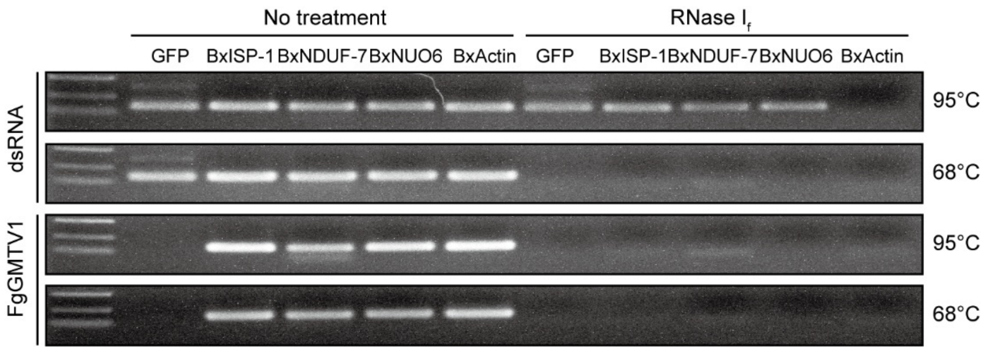

Figure S4. Detection of dsRNA in *B. xylophilus* after dsRNA soaking or fungal feeding. Total RNA was extracted from *B. xylophilus* after 2 days of dsRNA soaking or feeding on FgGMTV1-infected fungi carrying *B. xylophilus* gene fragments. The samples were subjected to RNase I_f treatment or left untreated. RNA was incubated at 95 °C for strong denaturing or at 68 °C for mild denaturing and followed by reverse transcription using gene-specific primers to synthesize the first cDNA strand. The resulting cDNA was used as a template for PCR amplification to detect dsRNA-derived transcripts. RT-PCR amplification of target fragments using RNAase I_f-treated RNA with denaturation at 95 °C but not at 68 °C confirms dsRNA nature of the RNA target.

Table S1. A list of primers used in this study.

Primers	Oligonucleotide sequence (5'- 3')	Purpose
FgGMTV1-BxISP-1-F	CAGCACTAACACGTGTaccggtGATTCTGTCCGGAGATACGC	To amplify *BxISP-1* gene, then clone into FgGMTV1
FgGMTV1-BxISP-1-R	GTATGTCCACCCAATCatgcatGGTGGCATCCTTAGTCGAAT	
FgGMTV1-BxNDUF-7-F	CAGCACTAACACGTGTaccggtGGATAGATATGGTGTAGTGTTCCG	To amplify *BxNDUF-7* gene, then clone into FgGMTV1
FgGMTV1-BxNDUF-7-R	GTATGTCCACCCAATCatgcatCACGATCCCATACTGATCAC	
FgGMTV1-BxNUO-6-F	CAGCACTAACACGTGTaccggtAAGCTCCAAATCAGAACATAAA	To amplify *BxNUO-6* gene, then clone into FgGMTV1
FgGMTV1-BxNUO-6-R	GTATGTCCACCCAATCatgcatCTCTGTAACGCCAAAGAACG	
FgGMTV1-GFP-F	CAGCACTAACACGTGTaccggtGCGAGGAGCTGTTCACC	To amplify GFP gene, then clone into FgGMTV1
FgGMTV1-GFP-R	GTATGTCCACCCAATCatgcatGCAGCTTGCCGGTGG	
FgGMTV1-212-F	GAAACTCACCCTACACTACTCCAAC	Universal primer of FgGMTV1-p26
FgGMTV1-212-R	CAGCGGAAATTTATTTTTAA	
FgGMTV1-Rep -F	CAGAGCACATACGAATCTCCA	Detection primers of FgGMTV1-Rep (DNA-A)
FgGMTV1-Rep -R	AGCGTCCATCCATTTCCAAT	
FgGMTV1-CP -F	CTGCCGAAACAACACCTTG	Detection primers of FgGMTV1-CP (DNA-B)
FgGMTV1-CP -R	CAGACGAAGAGGGACAACAT	
FgActin-F	ATCCACGTCACCACTTTCAA	Detection primers of *FgActin*
FgActin-R	TGCTTGGAGATCCACATTTG	
L4440-BxISP-1-F	CGAATTCCTGCAGCCCcccgggGATTCTGTCCGGAGATACGC	To amplify *BxISP-1* gene, then clone into L4440
L4440-BxISP-1-R	ACTAGTGGATCCACCGGTTCccatggGGTGGCATCCTTAGTCGAAT	
L4440-BxNDUF-7-F	CGAATTCCTGCAGCCCcccgggGGATAGATATGGTGTAGTGTTCCG	To amplify *BxNDUF-7* gene, then clone into L4440
L4440-BxNDUF-7-R	ACTAGTGGATCCACCGGTTCccatggCACGATCCCATACTGATCAC	
L4440-BxNUO-6-F	CGAATTCCTGCAGCCCcccgggAAGCTCCAAATCAGAACATAAA	To amplify *BxNUO-6* gene, then clone into L4440
L4440-BxNUO-6-R	ACTAGTGGATCCACCGGTTCccatggTCTGTAACGCCAAAGAACG	
L4440-GFP-F	CGAATTCCTGCAGCCCcccgggGCGAGGAGCTGTTCACC	To amplify GFP gene, then clone into L4440
L4440-GFP-R	ACTAGTGGATCCACCGGTTCccatggGCAGCTTGCCGGTGG	
L4440-F	TAATACGACTCACTATAGG	Universal primer of L4440
L4440-R	CCTATAGTGAGTCGTATTA	
qPCR-BxISP-1-F	TAATGCTGTTGTCTATGGAGTTG	To analyze expression level of *BxISP-1* by qPCR
qPCR-BxISP-1-R	GTAGATCCTTCAGGGACTTTAG	
qPCR-BxNDUF-7-F	TGCGATGTAAGCCGTTG	To analyze expression level of *BxNDUF-7* by qPCR
qPCR-BxNDUF-7-R	CAGCGTTTTCAGTTTCGGTA	
qPCR-BxNUO-6-F	AAAGCTCCAAATCAGAACAT	To analyze expression level of *BxNUO-6* by qPCR
qPCR-BxNUO-6-R	TCTGTAACGCCAAAGAACG	
qPCR-BxActin-F	GCAACACGGAGTTCGTTGTA	To analyze expression level of *BxActin* by qPCR
qPCR-BxActin-R	GTATCGTCACCAACTGGGAT	
dsRNA-BxISP-1-F	GATTCTGTCCGGAGATACGC	To analyze dsRNA expression level of *BxISP-1* by RT-PCR
dsRNA-BxISP-1-R	GGTGGCATCCTTAGTCGAAT	
dsRNA-BxNDUF-7-F	GGATAGATATGGTGTAGTGTTCCG	To analyze dsRNA expression level of *BxNDUF-7* by RT-PCR
dsRNA-BxNDUF-7-R	CACGATCCCATACTGATCAC	
dsRNA-BxNUO-6-F	AAGCTCCAAATCAGAACATAAA	To analyze dsRNA expression level of *BxNUO-6* by RT-PCR
dsRNA-BxNUO-6-R	CTCTGTAACGCCAAAGAACG	

dsRNA-GFP-F	GCGAGGAGCTGTTCACC	To analyze dsRNA expression level of GFP by RT-PCR
dsRNA-GFP-R	GCAGCTTGCCGGTGGTGCAGA	
